# Regeneration of Hair Cells in the Human Vestibular System

**DOI:** 10.3389/fnmol.2022.854635

**Published:** 2022-03-24

**Authors:** Yikang Huang, Huanyu Mao, Yan Chen

**Affiliations:** ^1^State Key Laboratory of Medical Neurobiology, Department of Otorhinolaryngology, Eye and ENT Hospital, MOE Frontiers Center for Brain Science, ENT Institute, Fudan University, Shanghai, China; ^2^NHC Key Laboratory of Hearing Medicine, Fudan University, Shanghai, China

**Keywords:** human, vestibule, hair cell, development, regeneration

## Abstract

The vestibular system is a critical part of the human balance system, malfunction of this system will lead to balance disorders, such as vertigo. Mammalian vestibular hair cells, the mechanical receptors for vestibular function, are sensitive to ototoxic drugs and virus infection, and have a limited restorative capacity after damage. Considering that no artificial device can be used to replace vestibular hair cells, promoting vestibular hair cell regeneration is an ideal way for vestibular function recovery. In this manuscript, the development of human vestibular hair cells during the whole embryonic stage and the latest research on human vestibular hair cell regeneration is summarized. The limitations of current studies are emphasized and future directions are discussed.

## Introduction

Vestibular sensory epithelia are composed of hair cells (HCs) and supporting cells (SCs). HCs of the vestibular sensory epithelia, which are surrounded by supporting cells, convert mechanical signals, such as head movement or tilt, into electrical signals (Gao et al., 2019; Liu et al., 2019; Qi et al., 2019, 2020; Tan et al., 2019). These signals are then transmitted by afferent fibers to the vestibular nuclei that send out fibers projected to the corresponding neural structures to control eye movement, posture, and balance (Cullen, 2012; Guo et al., 2019, 2020, 2021b,c; Hu et al., 2021; Wei et al., 2021).

Hair cells are easily injured by ototoxic drugs (Li et al., 2018; Zhang et al., 2019; Zhong et al., 2020; Fu et al., 2021b), aging (Cheng et al., 2019; Guo et al., 2021a; He et al., 2021), genetic factors (Qian et al., 2020; Cheng et al., 2021; Fu et al., 2021a; Lv et al., 2021; Zhang S. et al., 2021) and infections (Han et al., 2020; He et al., 2020; Zhang Y. et al., 2021). The loss of human vestibular HCs is closely related to balance dysfunction (Tsuji et al., 2000; Ishiyama et al., 2015). It has been stated that the annual incidence of vertigo is about 11% (Corrales and Bhattacharyya, 2016), and the lifetime prevalence of moderate to severe vertigo and dizziness is about 30% (Strupp et al., 2020). However, our current understanding of the development and generation of vestibular HCs is mainly derived from rodent models. Here, we review the current information on the development of human vestibular epithelia, as well as the latest progress made in restoring human vestibular HCs upon damage.

## Structure of Human Vestibular Sensory Epithelia

The human vestibular sensory epithelia, like that of other mammals, are composed of three crista ampullaris perpendicular to each other for sensing rotational motion of the head, and the utricular and saccular maculae, which detect linear acceleration (Angelaki and Cullen, 2008). In the mature state, the average surface areas of human cristae, utricular maculae and saccular maculae are around 0.9, 3.6, and 2.2 mm^2^, respectively (Watanuki and Schuknecht, 1976).

Hair cells are vestibular receptors located on sensory epithelia and surrounded by supporting cells. According to the different afferent synaptic terminals, human vestibular HCs can be further classified into two types: Type I HCs innervated by flask-shaped calyces and Type II HCs innervated by boutons (Wersall, 1956). There are several other morphological and functional differences between the two types which have been widely discussed in rodents (Rüsch et al., 1998), and the characteristics of these two types of HCs are similar in the human vestibule (Oghalai et al., 1998; Lim et al., 2014).

The human vestibular sensory epithelia can be divided into central and peripheral regions according to different characteristics. In the cristae, the central regions account for 46% of the total surface area. Type I HCs account for 70% of the central region HCs, while type II HCs account for 50% of the peripheral region HCs in the human cristae (Rosenhall, 1972a). In the utricular maculae, the central striola region accounts for about 8.6% of the surface area. The proportion of type I HCs in the striola region is higher and type II HCs show a high density in the peripheral region. The HC distribution in saccule maculae is similar to that of the utricle maculae (Rosenhall, 1972b).

Interestingly, the polarity of the hair bundle, which is determined by the position of kinocilia of HCs, varies between the human utricular and saccular maculae. The orientation of the utricular kinocilia is directed from the periphery toward the striola, while the kinocilia orientation is opposite in the saccule. Moreover, the striolar region of the utricular maculae is crescent, while the saccular maculae are “S” shaped (Rosenhall, 1972b).

Both the vestibular hair cell distribution and cilia polarity of humans are similar to those of mice. However, the number and differentiation time of vestibular HCs are significantly different between the two species, as will be discussed below.

## Development of Human Vestibular Epithelia

### Morphological Development of Human Vestibular Organs

**FIGURE 1:**
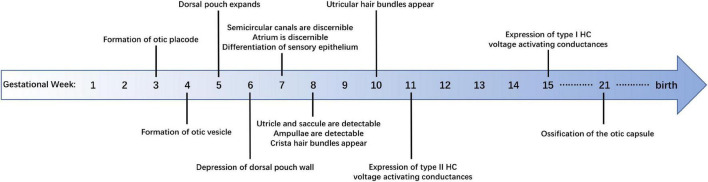
Summary of developmental milestones in the development of human vestibular sensory epithelia.

Anatomical studies have shown many details of human vestibular development (Figure 1). The formation of the otic placode is regarded as the first sign of inner ear development, which is the result of the ectoderm’s inner layer thickening at gestational week (GW) 3 (O’Rahilly, 1963). The otic placode then invaginates to form the otic cup that in turn pinches off the surrounding ectoderm and converts into the otic vesicle, composed of a dorsal (vestibular) and a ventral (cochlear) pouch, at the rhombomere 5 level by GW 4 (Streeter, 1906). From GW 4–5, the dorsal pouch expands into a triangular-shaped region forming the base of the three semicircular canals. The development of human anterior and posterior semicircular canals starts at embryonic days 41–43 with the depression of vestibular pouch wall, while the development of lateral semicircular canals begins a little later at embryonic days 44–46 (Yasuda et al., 2007). All semicircular canals are discernible at embryonic day (E) 47–E48 (Toyoda et al., 2015). Meanwhile, the atrium, which is the primordium of the utricle and saccule, can be observed in the ventral part of the vestibular pouch. Subsequently, a horizontal cleft that separates the atrium into an upper and lower compartment appears and the utricle and saccule are clearly detectable at E49–E51 (Streeter, 1906; Yasuda et al., 2007). By the end of the 5th month of the embryo, the bony labyrinth has been formed and the vestibular system is intact in morphology (Jeffery and Spoor, 2004), after which there is only a modest increase in the distance between the semicircular canals (Johnson Chacko et al., 2019).

Maturation of the human vestibular sensory epithelia includes lengthening of the cristae and thinning of the maculae. The length of cristae increases rapidly from GW 8–9 but slows down and changes in shape during GW 9–12. The anterior crista undergoes a second rapid growth during GW 12–14, reaching approximately 55% of adult size (Dechesne and Sans, 1985). The reduction of utricular epithelial thickness can be divided into two stages: GW 7–8 and GW 11–13, during which the number of supporting cell layers decreases significantly, while the thickness remains unchanged during GW 8–12. Finally, the supporting cell nuclei tend to be arranged in a single layer and the hair cell nuclei migrate to the cell base (Dechesne and Sans, 1985).

The morphogenesis of vestibular organs in humans is similar to that in rodents, but the human vestibular organs have been distinct in the early embryonic stage (by the end of GW 7, Figure 1; Streeter, 1906), while the mouse counterparts do not attain its mature shape until the late embryonic stage. The vestibular organs of mice become distinguishable on E15 and the membranous labyrinth morphology approaches maturity as late as E17 (Morsli et al., 1998).

### Differentiation of Vestibular Hair Cells

#### Cilia

Differentiation of human vestibular sensory epithelium does not occur until GW 7. The differentiation level of vestibular HCs is usually judged by their cilia, stereocilia and kinocilia.

Crista stereocilia are detectable at GW 8, during which time cristae are covered by short hair bundles with the putative kinocilia, the latter are longer than stereocilia in most cases and located at the edge of the bundle. Subsequently, there is a lengthening of cristae stereocilia, which leads to the longer and more mature hair bundles at GW10–11. From GW 12–14, the length of the hair bundles increases significantly, and the number of growing hair bundles decreased (Dechesne and Sans, 1985).

In the case of the utricle, the hair bundles observed at GW 10 are very short, and some kinocilia are shorter than the adjacent stereocilia. At GW 12, some hair bundles in the protruding utricle are close to maturity, while some are still growing. At GW 14, the hair bundles are morphologically mature and the number of newborn bundles decreases, while the microvilli are still abundant (Rosenhall and Engström, 1974; Dechesne and Sans, 1985), which indicates the HCs have not been fully developed so far.

From GW 14–18, hair bundles of HCs further mature in both human utricle and cristae. Stereocilia of the HCs in GW 18 fetus seem to be thicker than those in the GW 14 fetus. However, the number and diameter of stereocilia do not change significantly after GW 14, with about 80–100 stereocilia per HC (Rosenhall and Engström, 1974; Hoshino, 1982).

#### Molecular Marker

The differentiation of mammalian HCs is accompanied by the expression of cell-specific molecular markers, such as MyosinVIIa and Sox2. In the human vestibule, MyosinVIIa and Sox2 staining can be observed as early as GW 9. Moreover, the expression of MyosinVIIa is restricted to the vestibular HCs, while Sox2 is expressed in the supporting cells and a few of the HCs (Chacko et al., 2020).

In order to distinguish different cell types, recent work on mouse utricles has identified specific molecular markers for Type II HCs (Calretinin, Anxa4, and Mapt) and Type I HCs (Spp1 and Ocm) (McInturff et al., 2018). However, it remains to be investigated whether these vestibular hair cell markers are applicable to human specimens.

#### Electrophysiology

Evidences in electrophysiology demonstrate the functional similarities between human and rodent HCs. Whole-cell conductances of human vestibular HCs from GW 11–14 fetus are similar to those of mature type II HCs from rodents. The peak outward conductances obtained from human type II HCs increase from GW 15–18. Moreover, the rodent type I HC specific low-voltage activated K+ conductance, which is called G_K, L_, can also be detected in GW 15 human cristae, although relatively small (Lim et al., 2014). The similarities were further confirmed by the voltage-dependent currents that are expressed in vestibular HCs of both adult humans and rodents (Oghalai et al., 1998). However, so far there are few studies on the electrophysiological differences of vestibular HCs between humans and other mammals.

#### Hair Cell Number

There are no reports about the accurate time point at which the progenitors of human vestibular sensory epithelium begin to differentiate into hair cells since the spatio-temporal expression patterns of atonal homolog 1 (ATOH1), which is critical to HC formation, has not been investigated in the human vestibule so far. The distinct high expression of the HC marker, MyosinVIIa, is first observed in the crista as early as GW 9, indicating that some vestibular HCs have been formed at GW 9 (Johnson Chacko et al., 2020). But how these immature hair cells differentiate into type I and type II vestibular hair cells is not clear.

There is no significant difference in the number of cristae HCs between adults and the 4th–6th month fetuses: an average of 7,800 HCs per cristae at GW 16–23 and 7,500 HCs per cristae after birth (Rosenhall, 1972a). Another study observed an average of 8,005 HCs (type I 4,119 and type II 3,886) per lateral cristae of adults aged 26–67 years (Lopez et al., 2005).

It is reported that the number of HCs in the utricular and saccular maculae is about 2–4 times that in the cristae (Watanuki and Schuknecht, 1976). This study divided the specimens by age into the embryonic group (GW 14–23) and the postnatal group (<40 years old), and the average number of HCs was comparable between the two groups. As for the utricle, the average number is 33,100 (2,300 in the central area), with 32,900 HCs in the embryonic group and 33,200 in the postnatal group. The average number of saccular maculae HCs is 18,800 (1,600 in the central area), with 19,100 in the embryonic group and 18,400 in the postnatal group. Another study about utricles shows that the average number of utricular HCs at GW 16 is about 36,000, not significantly different from that at the age of 15, but significantly higher than the 13,000 at GW 10–12 (Severinsen et al., 2010).

Overall, the time point of HC differentiation is remarkably earlier in humans than that in rodents. As mentioned above, the number of human vestibular HCs reaches the adult level no later than the 5th month of gestation. In contrast, over half the mouse HC population is formed after birth (Burns et al., 2012).

### Factors Related to the Development of Human Vestibular Sensory Epithelia

Several reviews have summarized the relevant regulatory factors in hair cell development in animal models. In general, mammalian hair cell development involves the emergence of Sox2-labeled pro-sensory areas, the expression of transcription factor Atoh1, the regulation of cell cycle by factors such as p27^*Kip*1^, and the manipulation by signaling pathways such as Notch, Fgf, Wnt, Shh, and Bmp (Wu and Kelley, 2012; Atkinson et al., 2015; Whitfield, 2015). However, there haven’t been many studies on the regulation of human hair cell development so far.

Proliferation and apoptosis are essential processes during human inner ear development. It is demonstrated by Ki-67 staining that the percentages of proliferating cells in the utricle and semicircular canal are 43 and 38%, respectively, at GW 6, but decrease to 24 and 30% at GW 9. However, the trend of Bcl-2 expression in the vestibular epithelium is opposite to that of Ki67 during GW 7–10. Moreover, cysteine aspartate-specific protease-3 (caspase-3) and insulin-like growth factor-1 (IGF-1) are also expressed during vestibular epithelial development (Tafra et al., 2014). These results suggest that factors related to proliferation and apoptosis may contribute to the morphogenesis and differentiation of vestibular sensory epithelia.

Brain-derived neurotrophic factor (BDNF) is a neurotrophic protein. Previous studies have shown that BDNF plays an important role in vestibular nerve development in animals (Fritzsch et al., 1997). In the human vestibule, BDNF is firstly expressed in the entire utricular sensory epithelium, but its expression decreases from GW 9–12 and is restricted in the extrastriola at GW 12. In adult human utricles, BDNF is only present in the apical part of HCs. The expression of p75NTR in vestibular organs and TrkB and C in nerve fibers increase with development, suggesting an essential role of neurotrophic receptors in the survival of vestibular neurons during early embryonic stages (Johnson Chacko et al., 2017).

Another study reported the expression of several key transcription factors during human inner ear development. For the vestibular sensory epithelia, LGR5 expression increased from GW 8–12 and was broad in the apical poles of the vestibular HCs. Another transcription factor, GATA3, was expressed in the striola of the utricular and saccular maculae at GW 11. Expression for SOX2 was primarily restricted to the utricular supporting cells at GW 9, suggesting its function in regulating the differentiation of supporting cells (Johnson Chacko et al., 2020). These results indicate that the active transcription factors during the development of the mammalian inner ear may also play a critical role in the development of human vestibular sensory epithelia. However, the spatio-temporal expression patterns of other genes essential for hair cell formation, such as Math1, Six1, Gfi1, and Pou4f3, are not explored in the human vestibule up to now.

In general, research on vestibules of human embryos is quite limited and the previous work mainly focused on the expression of specific molecules. Further experiments are required for demonstrating the similarities and differences of vestibular hair cell development between human and animal models, and the underlying mechanisms as well.

## Regeneration of Human Vestibular Hair Cells

### Discovery of Hair Cells Regeneration in the Mammalian Vestibular Epithelium

Considering that no artificial device can be used to replace vestibular function, recovery from vestibular dysfunction mainly depends on the compensation of central vestibular function, which can hardly lead to a full recovery. Promoting vestibular HC regeneration is an ideal way for vestibular function recovery.

Many studies have shown differences in the regeneration ability of vestibular sensory epithelium between different species. Non-mammalian vertebrates such as birds are able to produce HCs throughout their lives (Balak et al., 1990; Roberson et al., 1992; Weisleder and Rubel, 1992). In contrast, the restoration of vestibular HCs is relatively limited in mammals (Forge et al., 1993; Wang et al., 2015; Wu et al., 2016; You et al., 2018; Zhang et al., 2020), which can be realized through two processes, namely, mitosis or trans-differentiation (Rubel et al., 1995).

Loss of vestibular HCs, whether induced by aminoglycoside antibiotics (Kawamoto et al., 2009), IDPN (Zeng et al., 2020) or other injury methods (Golub et al., 2012; González-Garrido et al., 2021), significantly enhances spontaneous regenerative proliferation. However, compared to the complete recovery of vestibular function in most non-mammalian vertebrates (Jones and Nelson, 1992; Carey et al., 1996), both the number and the function of the newborn HCs are limited in the mammalian vestibular epithelium (Forge et al., 1993; Kawamoto et al., 2009; Golub et al., 2012; Zeng et al., 2020; González-Garrido et al., 2021). As a result, techniques to boost the regeneration of mammalian vestibular HCs are needed.

### Manipulation of Vestibular Hair Cell Regeneration in Mammals

Considering the important role of growth factors in the development of the mammalian inner ear, many studies want to reveal whether they could also initiate the generation of vestibular HCs. Through *in vitro* culture, transforming growth factor alpha (TGF-α) is first found to be capable of restoring HCs in adult mouse vestibular organs after injury (Lambert, 1994). Subsequently, epidermal growth factor (EGF) (Yamashita and Oesterle, 1995), fibroblast growth factor (FGF) family members, IGF-1 and IGF-2 (Zheng et al., 1997) are demonstrated to trigger the proliferation of rat vestibular epithelial cells together with TGF-α. Recombinant human glial growth factor 2 (rhGGF2) and insulin are also capable of evoking great cell proliferation in the utricular epithelium of neonatal rats (Gu et al., 2007). Compared to TGF-α alone, simultaneous infusion of TGF-α and insulin into the rat inner ear shows combined effects in producing HCs (Kuntz and Oesterle, 1998a,b). Furthermore, combined utilization of TGF-α, IGF-1, and retinoic acid (RA) and BDNF performs well in restoring type I vestibular HCs *in vivo* and suggests application value (Kopke et al., 2001).

Regulation of intracellular signals is another method of promoting regeneration. Activation of phosphatidylinositol-3 kinase (PI-3K), mammalian target of rapamycin (mTOR), protein kinase C (PKC), mitogen-activated protein kinase (MAPK) and increased intracellular calcium enhance the proliferation of cells in murine vestibular epithelia. All of these signals are closely associated with the S-phase entry (Montcouquiol and Corwin, 2001).

The basic helix-loop-helix (bHLH) transcription factor, atonal homolog 1 or Atoh1, is critical for the differentiation of HCs (Bermingham et al., 1999). Atoh1 overexpression activates the HC differentiation in murine vestibular epithelia both *in vitro* (Zheng and Gao, 2000; Huang et al., 2009; Qian et al., 2021) and *in vivo* (Staecker et al., 2007; Schlecker et al., 2011; Gao et al., 2016; Sayyid et al., 2019), which can be enhanced by injury (Staecker et al., 2007; Schlecker et al., 2011; Sayyid et al., 2019; Hicks et al., 2020; Qian et al., 2021) and depressed by aging (Gao et al., 2016). On the other hand, Atoh1 deletion inhibits the spontaneous returning of HCs significantly (Hicks et al., 2020).

Disrupting the lateral inhibition established by Notch signaling is another classic strategy for producing new HCs. After adding DAPT and TAPI-1, two inhibitors of the Notch signaling pathway, to the explanted utricles of adult mice, enhanced hair cell regeneration was observed, especially in the striolar/juxtastriolar region (Lin et al., 2011). DAPT treatment also leads to extensive HC generation in cristae explants of adult mice (Slowik and Bermingham-McDonogh, 2013). Moreover, downregulation of the Notch target gene Hes5 through siRNA can also induce the trans-differentiation of supporting cells and boost hair cell regeneration in the damaged mouse utricles (Jung et al., 2013).

Apart from the traditional means mentioned above, recent studies have revealed some new targets for regulating HC regeneration. Several studies connected the ability of supporting cells to reenter the cell cycle during murine utricular development or after injury to nuclear Yap signaling (Gnedeva et al., 2017, 2020; Kastan et al., 2021). Collado et al. (2011) found the accumulation of E-Cadherin as an inhibitor for trans-differentiation of supporting cells. Knockdown of Foxg1 in supporting cells was revealed to be another viable method of enhancing HC regeneration in the neonatal mouse utricle (Zhang et al., 2020). In order to achieve better regenerative effects, attempts have been made to simultaneously promote supporting cells proliferation and hair cell differentiation through a combined regulation of multiple signaling pathways, such as Wnt and Notch (Wu et al., 2016).

Although vestibular hair cell regeneration in mammals still faces many hurdles, the good news is that murine vestibular function, under certain modulations, has been partially restored after injury (Kopke et al., 2001; Staecker et al., 2007; Schlecker et al., 2011). However, whether the regeneration phenomenon can also be discovered in humans and whether it can be regulated in the same manner as in rodents are questions that must be addressed for the clinical application of regenerative techniques.

### Regenerative Potential of Human Vestibular Hair Cells

Studies on the regenerative potential of human vestibular HCs started almost at the same time as that of other mammals. Through *in vitro* experiments, Warchol et al. (1993) found proliferating supporting cells after neomycin injury in the human utricle. After 25 days of culture, some labeled nuclei in the lumenal stratum that was normally occupied by the nuclei of HCs appeared, which suggested the restoration of HCs (Warchol et al., 1993). In another study, 22 patients with Meniere’s disease were tested on their vestibular function 1–2 years after gentamicin treatment. The horizontal semicircular canal afferent nerve restored its excitability to warm and cold water in 38% of them, which was regarded as functional evidence for HC regeneration (De Waele et al., 2002). However, the evidence here is insufficient, in which the study could not confirm the direct cause of the functional recovery, since vestibular inhibition and central compensation could also lead to functional restoration. The morphological evidence came in 2015. Immature hair bundles were observed in human vestibular specimens harvested from elderly patients (Taylor et al., 2015). Given that no evidence of the hair bundle restoration has been detected on surviving “bald” HCs, there is likely to be spontaneous HC regeneration occurring in human utricles.

Another approach to investigate the stemness of vestibular sensory epithelial cells is to extract progenitors or stem cells from the human inner ear. It was first realized by Chen et al. (2009) who extracted stem cells capable of differentiating into HCs from the fetal cochlea in 2009. Since then, attempts have been made in vestibular organs. Hu et al. (2012) isolated sensory epithelial cells from postoperative human utricular specimens and cultured them *in vitro*. The proliferated cells expressed genes in pre-sensory cells or stem cells such as SOX2 and P27^*KIP*1^ and showed characteristics of mesenchymal cells when cultured on 2D substrates (Hu et al., 2012). A recent study demonstrated that the human vestibular epithelial cells have a strong sphere-forming ability through *in vitro* culture. Furthermore, the clonal spheres were able to produce cells expressing markers of HCs and displayed differentiation ability (Senn et al., 2020). The existence of multipotent progenitor cells in the adult human vestibule indicates the capability of sensory epithelial cells to re-enter the cell cycle and provides a promising source for neonatal HCs.

In conclusion, the regenerative potential of human vestibular HCs has been revealed from different aspects, reflecting the similarities between human and rodent vestibules. Therefore, the theoretical basis for the regulation of human hair cell regeneration following the example of other mammals has been established.

### Application of Gene Therapy in Human Vestibular Sensory Epithelia

As is mentioned above, overexpression of Atoh1 serves as a classic approach to triggering regeneration of vestibular HCs in rodents. In 2003, Shou et al. (2003) upregulated HATH1 (a human homolog of Atoh1) in cultured adult rat utricular maculae through local adenoviral treatment and robust production of new HCs was observed in normal and gentamicin-injured utricles as a result of supporting cell conversion, implying the conserved function of the atonal homologs during the revolution. Moreover, considering the similarity between human and murine homologs of atonal in giving rise to HCs, overexpressing HATH1 may be a promising target for gene therapy in human balance organs.

Choosing a suitable transduction vector is essential for inner ear gene therapy. Adenovirus has been demonstrated to efficiently transfect both HCs and supporting cells, thus becoming a competitive candidate. In 2007, Kesser et al. (2007) developed a multi-gene deletion and replication-free adenovirus vector (AD2) to test its transfection efficacy in human tissues, which drove the expression of the green fluorescent protein GFP gene (AD2-GFP) by cytomegalovirus (CMV) promoter. Results indicated that both supporting cells and HCs were transfected and the transfection rate was higher in supporting cells and varied with viral titer and transfection time. Furthermore, the adenovirus vector also performed well when GFP and wild-type potassium channel gene KCNQ4 were transfected simultaneously into the human inner ear, with 17.3% hair cell transfection rate for GFP and 10% for KCNQ4 (Kesser et al., 2007). Adeno-associated virus (AAV) is another promising vector that has enabled efficient gene transfer to several organs. Recently, an AAV variant (AAV-ie) has been designed for inner ear gene delivery. It was shown that AAV-ie infected about 93% of SCs and 76% of HCs in human utricle. In addition, the saccular macula and cristae could be transduced as well (Tan et al., 2019).

Based on the maturity of the transduction vector, Taylor et al. (2018) made the first attempt to generate HCs in human utricle. Taylor et al. (2018) collected utricles from patients undergoing excision of vestibular schwannoma and HCs were ablated through gentamicin. Ad2-GFP-Atoh1 was used here to transfect utricular maculae and supporting cells were efficiently transduced. Compared with the control group, transfection successfully increased the HC number in the maculae. Moreover, Notch signaling pathway inhibitor TAPI-1 also induced regeneration in human utricles, while the newborn HCs were fewer than those in the Atoh1 transfection group. Finally, no synergistic effect of the two treatments was observed, implying Atoh1 overexpression as a more effective solution. This study creates a precedent for gene therapy targeting the human inner ear.

## Future Perspectives

Although gene therapy targeting human vestibular epithelium has triggered HC regeneration successfully, many problems remain to be addressed before more mature and functional HCs can be generated. Considering the similarities between vestibular and auditory sensory epithelium, recent studies on the cochlea may offer some inspiration.

Atoh1-induced HC regeneration still has some limitations in both mouse and human vestibules. First, the number of hair cells could not be fully restored after injury. Second, nearly all the new hair cells converted from supporting cells were Sox2 + type II hair cells, the lack of new type I hair cells is not conducive to vestibular rehabilitation. Moreover, the regenerated hair cells were immature, as demonstrated by immature cilia (Schlecker et al., 2011; Taylor et al., 2018; Sayyid et al., 2019; Qian et al., 2021). Recent studies report some progresses in cochlear hair cell regeneration by manipulating multiple signaling pathways or transcriptional factors (Walters et al., 2017; Lee et al., 2020; Menendez et al., 2020; Chen et al., 2021; Sun et al., 2021). In order to improve both the quality and quantity of new vestibular hair cells, regulating multiple factors which can promote the maturation and subtype differentiation of vestibular hair cells may be an important strategy. In addition, it might be beneficial to combine epigenetic regulatory factors with Atoh1 overexpression to promote hair cell regeneration in the human vestibule.

Generating organoids provides ideal models for screening drug candidates for the treatment of inner ear diseases. Recent studies have succeeded in producing otic organoids from human pluripotent stem cells. Some of them shared many characteristics with human vestibular epithelia (Koehler et al., 2017; Jeong et al., 2018; van der Valk et al., 2021). In the future, exploring the regulatory mechanisms underlying hair cell formation using organoids and the latest techniques, such as single-cell and single-nuclear sequencing, may help to identify regulatory pathways and key factors which play important roles in the maturation and subtype differentiation of vestibular hair cells. Progresses in this area will contribute to the study of hair cell regeneration in human vestibule and balance function reconstruction.

Chen et al. (2012) transplanted otic neuroprogenitors derived from human embryonic stem cells (hESCs) into ouabain-treated gerbils (an auditory neuropathy model) through the round window, and restoration of auditory evoked response was observed. The protocol of transplanting progenitor cells discussed here offers another promising strategy for generating functional HCs in the human vestibule.

Trans-differentiation of Lgr5+ or Plp1+ supporting cells is traditionally regarded as the main source of postnatal hair cell generation in utricles (Wang et al., 2015, 2019). However, the latest studies suggested transitional epithelial cells (TECs) located at the border between sensory and non-sensory regions to be another reliable source of HCs (Huang et al., 2009; Burns et al., 2015; Gao et al., 2016; Jan et al., 2021; Qian et al., 2021). How to achieve effective trans-differentiation from TECs into supporting cells or HCs could be another future direction.

## Author Contributions

YH, YC, and HM wrote and revised the manuscript. All authors contributed to the article and approved the submitted version.

## Conflict of Interest

The authors declare that the research was conducted in the absence of any commercial or financial relationships that could be construed as a potential conflict of interest.

## Publisher’s Note

All claims expressed in this article are solely those of the authors and do not necessarily represent those of their affiliated organizations, or those of the publisher, the editors and the reviewers. Any product that may be evaluated in this article, or claim that may be made by its manufacturer, is not guaranteed or endorsed by the publisher.

## Abbreviations

AAV: adeno-associated virus
AD: adenovirus
Anxa4: annexin A4
Atoh1: atonal homolog 1
BDNF: brain-derived neurotrophic factor
bHLH: basic helix-loop-helix
caspase-3: cysteine aspartate-specific protease-3
CMV: cytomegalovirus
E: embryonic day
EGF: epidermal growth factor
FGF: fibroblast growth factor
GATA3: GATA binding protein 3
GW: gestational week
HATH1: human homolog of Atoh1
HC: hair cell
hESCs: human embryonic stem cells
IDPN: 3,3′-iminodiproprionitrile
IGF-1: insulin-like growth factor-1
IGF-2: insulin-like growth factor-1
LGR5: leucine-rich repeat-containing G protein-coupled receptor 5
MAPK: mitogen-activated protein kinase
Mapt: microtubule associated protein
mTOR: mammalian target of rapamycin
Ocm: oncomodulin
PI-3K: activation of phosphatidylinositol-3 kinase
PKC: protein kinase C
RA: retinoic acid
rhGGF2: recombinant human glial growth factor 2
SC: supporting cell
SOX2: SRY (sex-determining region Y)-box 2
Spp1: secreted phosphoprotein 1
TGF-α: transforming growth factor alpha.
